# Lessons from LIMK1 enzymology and their impact on inhibitor design

**DOI:** 10.1042/BCJ20190517

**Published:** 2019-11-05

**Authors:** Eidarus Salah, Deep Chatterjee, Alessandra Beltrami, Anthony Tumber, Franziska Preuss, Peter Canning, Apirat Chaikuad, Petra Knaus, Stefan Knapp, Alex N. Bullock, Sebastian Mathea

**Affiliations:** 1 Target Discovery Institute, Nuffield Department of Medicine, University of Oxford, Roosevelt Drive, Oxford OX3 7FZ, U.K.; 2 Structural Genomics Consortium, University of Oxford, Old Road Campus, Roosevelt Drive, Oxford OX3 7DQ, U.K.; 3 Institute of Pharmaceutical Chemistry, Goethe-University Frankfurt, 60438 Frankfurt, Germany; 4 Structural Genomics Consortium, BMLS, Goethe-University Frankfurt, 60438 Frankfurt, Germany; 5 Institute for Chemistry and Biochemistry, Freie Universität Berlin, 14195 Berlin, Germany

**Keywords:** kinase, LIMK1, reaction mechanism, small-molecule inhibitors, substrate recognition

## Abstract

LIM domain kinase 1 (LIMK1) is a key regulator of actin dynamics. It is thereby a potential therapeutic target for the prevention of fragile X syndrome and amyotrophic lateral sclerosis. Herein, we use X-ray crystallography and activity assays to describe how LIMK1 accomplishes substrate specificity, to suggest a unique ‘rock-and-poke’ mechanism of catalysis and to explore the regulation of the kinase by activation loop phosphorylation. Based on these findings, a differential scanning fluorimetry assay and a RapidFire mass spectrometry activity assay were established, leading to the discovery and confirmation of a set of small-molecule LIMK1 inhibitors. Interestingly, several of the inhibitors were inactive towards the closely related isoform LIMK2. Finally, crystal structures of the LIMK1 kinase domain in complex with inhibitors (PF-477736 and staurosporine, respectively) are presented, providing insights into LIMK1 plasticity upon inhibitor binding.

## Introduction

Fragile X syndrome (FXS) is a genetic disorder caused by a mutation in the *Fragile X mental retardation 1* (*FMR1*) gene [1]. Affected individuals show developmental delay and reduced intellectual abilities, often paired with autism, social anxiety and hyperactivity [1]. With a prevalence of 1 in 6000 newborns, FXS is the most common hereditary cause of intellectual disability [1]. Amyotrophic lateral sclerosis (ALS) is characterised by the impaired formation of motor neurons [2]. In affected individuals, the gradual loss of muscle functionality leads to difficulties in speaking, movement and breathing [2]. ALS etiology is complex being associated with both genetic and environmental factors. However, the most prominent genetic risk factor is a mutation in the *C9ORF72* gene, accounting for ∼5% of cases [3]. ALS is classified a rare disease, with 2.6 in 100 000 people per year being diagnosed with ALS in Europe [4]. As for FXS, there is no known cure [2]. Effective treatments are urgently sought for both diseases.

While FXS and ALS differ in trigger, affected cell type, age of onset and clinical appearance, both disorders share a common cellular characteristic in the deregulation of actin cytoskeleton dynamics [5,6]. A deregulated cytoskeleton impairs multiple cellular functions such as motility, neurite growth and vesicle transport. Actin-depolymerising factors (ADFs), namely cofilin-1 (CFL1), cofilin-2 (CFL2) and destrin, are key regulators of actin cytoskeleton dynamics [7]. These small proteins with high sequence identity decorate ADP-rich segments of actin filaments (generally the older segments), thus promoting filament severing and disassembly [7]. This provides the cell with fresh ATP-actin monomers with which to build new actin filaments as they are required.

The ADF activity, in turn, is regulated by phosphorylation, with several kinases inactivating, and the phosphatase Slingshot homologue 1 (SSH1) [8] activating ADFs. The kinases capable of phosphorylating ADFs belong to the tyrosine kinase-like family of protein kinases and include the LIM domain kinases 1 and 2 (LIMK1 and LIMK2) [9] and the testis-specific kinases 1 and 2 (TESK1 and TESK2) [10]. The contribution of an individual kinase to ADF phosphorylation is cell type-specific, developmental stage-specific and thus difficult to establish. In adult neurons, however, LIMK1 is regarded as the dominant factor for ADF phosphorylation. LIMK1 accounts for 70%, LIMK2 for 15% and TESK1/2 for the residual 15% of Phospho-ADFs, as determined by analysing the hippocampi of knockout mice [11].

**Figure 1.**
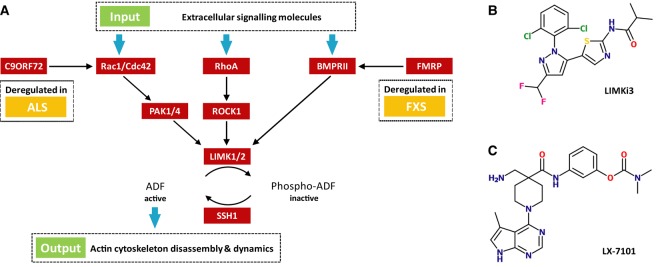
The ADF cascade regulates actin cytoskeleton dynamics. (**A**) The ADF cascade regulates actin cytoskeleton dynamics. Several pathways converge in LIMK1/2. In both ALS and FXS, the cascade is deregulated. (**B**) Chemical structure of LIMKi3, a widely used LIMK1 inhibitor characterized by high LIMK1 affinity, but unfavourable off-target activity [17]. (**C**) Chemical structure of LX-7101, a dual inhibitor targeting LIMK1 and ROCK1 [20].

LIMK1/2 activity is switched on by upstream kinases such as p21-activated kinase 1 and 4 (PAK1 and PAK4), Rho-associated protein kinase 1 (ROCK1) and bone morphogenetic protein (BMP) receptor type-2 (BMPR2) [12]. These kinases, in turn, are regulated by Rho-family GTPases or directly by growth factors such as the BMPs [12]. Thus, multiple signals converge on LIMK1/2, are integrated and translated into the ADF phosphorylation level. Important aspects of the complex, but well-established ADF cascade are depicted in Figure 1A.

Due to its crucial role in the phosphorylation of neuronal ADFs, LIMK1, in particular, has been identified as a promising therapeutic target for the prevention of both FXS [13,14] and ALS [15]. Small-molecule inhibition of LIMK1 kinase activity is expected to compensate for the effects of mutated *FMR1* [13] and *C9ORF72* [15], respectively. Several LIMK1 inhibitors have been developed [16], most notably the now widely used LIMKi3 [17] (Figure 1B). The thiazole derivative shows high potency for LIMK1 (*in vitro* IC_50_ = 7 nM) and reasonable selectivity against the kinome with 5′-AMP-activated protein kinase (AMPK) being the most prominent kinase off-target [17]. The inhibitory activity of LIMKi3 towards LIMK1 was also demonstrated in several cancer cell lines [18] and in authentic prostate tissue [19], where a reduction in CFL1 phosphorylation was observed. However, LIMKi3 is of limited use in biological settings, since it strongly interacts with tubulin [17]. The pyrrolopyrimidine LX-7101 [20] (Figure 1C) is another potent LIMK1 inhibitor (IC_50_ = 32 nM) with more moderate kinome-wide selectivity. Notably, the favourable pharmacological properties of LX-7101 led to its investigative use in phase I clinical trials for glaucoma [20]. Other LIMK1 inhibitors are less well characterized [21]. However, we are not aware of any LIMK1 inhibitor that does not inhibit LIMK2 with similar affinity. This is not surprising, since LIMK1 and LIMK2 share high sequence identity within their kinase domains (71%), with the ATP-binding pocket residues being close to identical.

Both LIMK proteins exhibit the same domain architecture and have overlapping substrate specificities. Although LIMK1 is more highly expressed in the brain and LIMK2 is more abundant in muscle tissue, most tissues express both proteins (data from The Human Gene Atlas). Nonetheless, different regulatory mechanisms and physiological roles are apparent as reflected in their knockout phenotypes in mice. In contrast with LIMK1, LIMK2 is indispensable for the proper functionality of the eye [22] and in spermatogenesis [23]. Therefore, isoform-specific LIMK inhibitors would be valuable tools, both for deciphering the physiological roles of the individual LIMK isoforms and for the validation of LIMK1 as a target for the treatment of FXS and ALS. Ideally, these inhibitors will also serve as starting points for the development of therapeutics.

Many aspects of LIMK1 structure and function are still to be completely characterised. A significant development was the recent publication of a 3.5 Å model of the LIMK1–CFL1 complex [24]. However, there remain open questions regarding LIMK1's substrate specificity and as yet there have been no published structural models of LIMK1-inhibitor complexes. Here we show that LIMK1 robustly phosphorylates both serine and tyrosine residues, establishing LIMK1 as a dual-specificity kinase. We also present a higher resolution structure of the LIMK1–CFL1 complex as well as the first structures of two LIMK1-inhibitor complexes. Finally, we propose a new rock-and-poke mechanism for substrate phosphorylation by the LIM kinases.

## Experimental

### Compound libraries and peptides

The kinase inhibitor library L1200 and the inhibitors for activity assays and co-crystallisation were purchased from Selleckchem. The peptides tested as LIMK1 substrates were purchased from Genscript (description in Supplementary Table S2).

### LIMK1_KD_ and LIMK2_KD_ cloning, expression and purification

The cDNA coding for LIMK1 residues 330 to 637 was PCR amplified using the forward primer TACTTCCAATCCATGCCACACCGCATCTTCCG and the reverse primer TATCCACCTTTACTGTCAGCTCTCGCCGCGCCGG. The cDNA coding for LIMK2 residues 330 to 632 was PCR amplified using the forward primer TACTTCCAATCCATGGACCTAATCCATGGGGAGGTCCTGG and the reverse primer TATCCACCTTTACTGTCACAGGCCGTACTGCATGCTCACAG. Both PCR templates were taken from the Mammalian Gene Collection (MGC). The PCR products were then inserted into the vector pFB-LIC-Bse via ligation independent cloning (LIC) [25]. After the transposition of the coding sequence into an engineered Baculovirus genome (Bac-to-Bac, Invitrogen), the viral DNA was transfected into Sf9 insect cells cultivated in InsectXpress medium (Lonza). Protein expression was performed as previously described [26]. In brief, exponentially growing TriEx cells (Novagen) at 2 × 10^6^ cells/ml were infected 1 : 64 with virus stock, incubated for 66 h at 27°C under constant shaking and harvested by centrifugation.

Cells were resuspended in lysis buffer (50 mM HEPES pH 7.4, 500 mM NaCl, 20 mM imidazole, 0.5 mM TCEP, 5% glycerol) and lysed by sonication. The lysate was cleared by centrifugation and loaded onto a Ni NTA column. After vigorous rinsing with lysis buffer, the His_6_-tagged proteins were eluted in lysis buffer containing 300 mM imidazole. While the proteins were subjected to dialysis to get rid of the imidazole, the N-terminal tags were cleaved by TEV protease. Contaminating proteins, the cleaved tags and TEV protease were removed with another Ni NTA step. Finally, the LIMK kinase domains were concentrated and subjected to gel filtration using an AKTA Xpress system combined with an S200 16/600 gel filtration column (GE Healthcare). The elution volumes of 91.8 ml (LIMK1_KD_) and 91.6 ml (LIMK2_KD_) indicated the proteins to be monomeric in solution. The final yields were 2.0 mg/L insect cell medium (LIMK1_KD_) and 0.2 mg/L insect cell medium (LIMK2_KD_).

### CFL1 cloning, expression and purification

cDNA coding for CFL1 (again taken from MGC) was PCR amplified using the forward primer TTAAGAAGGAGATATACTATGGCCTCCGGTGTGGCTGTC and the reverse primer GATTGGAAGTAGAGGTTCTCTGCCAAAGGCTTGCCCTCCAGGG. The PCR product was then inserted into the vector pNIC-CTHF via ligation independent cloning [25]. The mutants S3C, S3T, S3Y, K112A, K112E, A118P and A118W were generated by site-directed mutagenesis using the QuikChange kit (Agilent). The resulting plasmids were transformed into *E. coli* DH10B, protein expression was performed as previously described [27]. In brief, bacteria were propagated in TB medium at 37°C until the OD reached 1. Under constant shaking, the temperature was then reduced to 18°C. After 30 min, IPTG was added to a final concentration of 0.5 mM. The bacteria were incubated for another 16 h at 18°C and harvested by centrifugation.

Cells were resuspended in lysis buffer (50 mM HEPES pH 7.4, 500 mM NaCl, 20 mM imidazole, 0.5 mM TCEP, 5% glycerol) and lysed by three passages through the high-pressure cell breaker. The purification was carried out as described above for LIMK1_KD_. In the final gel filtration with the S75 16/600 column (GE Healthcare), CFL1 eluted at 78.7 ml, indicating the protein to be monomeric in solution. The final yield was 70 mg CFL1/L TB medium.

### Dynamic scanning fluorimetry (DSF) assay

The assay was performed according to a previously established protocol [28]. A solution of 2 µM LIMK1_KD_ in assay buffer (20 mM HEPES pH 7.4, 150 mM NaCl, 0.5 mM TCEP, 5% glycerol) was mixed 1 : 1000 with the SYPRO Orange dye (Sigma). The compounds to be tested were added to a final concentration of 10 µM. An amount of 20 µl of each sample were placed in a 96-well plate and the temperature increased from 25 to 95°C. Fluorescence was monitored using a Mx3005P real-time PCR instrument (Stratagene) with excitation and emission filters set to 465 and 590 nm, respectively. Data were analysed with the MxPro software. The screening was performed as a single shot experiment. Potential LIMK1_KD_ binders were further analysed in an activity assay for confirmation.

### Rapidfire mass spec kinase activity assay

RapidFire mass spectrometry offers a high-throughput, label-free and direct measurement of substrate modifications, including protein phosphorylation. This technology couples desalting by solid-phase extraction (SPE) to rapid automated sample injection and processing to achieve high sampling rates. For inhibitor IC_50_ determination, the respective inhibitors in DMSO (11-point concentration series, all points in technical duplicates) were dispensed to a 384-well polypropylene plate using an ECHO 550 acoustic dispenser (Labcyte). Then, LIMK1_KD_ was added (final concentration 40 nM) to allow for 10 min pre-incubation at room temperature. The phosphorylation reaction was initiated by adding a mixture of CFL1 (final concentration 2 µM) and ATP (final concentration 800 µM). After 60 min incubation at room temperature, the reaction was stopped by adding formic acid to a final concentration of 1%. The reaction volume was 50 µl, and the assay buffer composition was 50 mM Tris pH 7.5, 0.1 mM EDTA, 0.1 mM EGTA, 1 mM MgCl_2_.

The plate was transferred to a RapidFire RF360 high-throughput sampling robot (Agilent). Samples were aspirated under vacuum and loaded onto a C4 SPE cartridge and washed for 5.5 s with 0.1% (v/v) formic acid in LCMS grade water to remove non-volatile buffer components. After the aqueous wash, analytes of interest were eluted from the C4 SPE onto a 6530 Q-TOF LC/MS (Agilent) in an organic elution step (85% acetonitrile in LCMS grade water containing 0.1% formic acid). Ion data for the CFL1 substrate and the Phospho-CFL1 product was extracted and peak area data integrated using RapidFire integrator software (Agilent). Finally, IC50 curves were generated using GraphPad Prism 7.

### Crystallisation

Initially, the crystallisation of each sample was explored with 384 diverse precipitant solutions (four coarse screens, namely HCS3, HIN3, JCSG7 and LFS6 purchased from Molecular Dimensions). Promising conditions were optimised by modulating the concentrations, the pH and the volume of the respective precipitant solution. To obtain mountable crystals, 100 nL-drops of the protein solution with the respective ligand were transferred to a 3-well crystallisation plate (Swissci), mixed with 50 nL precipitant solution and incubated at 4°C (see Table 1 for full protein, ligand and precipitant details). Crystals appeared overnight and did not change appearance after 7 days. They were mounted in precipitant solution cryoprotected with additional 25% ethylene glycol. Data were collected at Diamond Light Source, and analysed, scaled and merged with Xia2 [29]. The structures were solved by molecular replacement with Phaser [30] using a Src model (PDB ID 1YI6) as a template for LIMK1 and a CFL1 model (PDB ID 4BEX) as a template for CFL1. The models were refined with Refmac5 [31] and validated using MolProbity [32]. A summary of data collection and refinement statistics is given in Table 1. The models and structure factors have been deposited with the PDB IDs 5NXC, 3S95 and 5L6W, respectively.

**Table 1:** Crystallisation conditions, diffraction data collection and refinement statistics

PDB ID	5NXC	3S95	5L6W
Protein solution	10 mg/ml LIMK1_KD_	8.0 mg/ml LIMK1_KD_	6.5 mg/ml LIMK1_KD_, 3.5 mg/ml CFL1
With ligand	500 µM PF-477736	500 µM staurosporine	1.2 mM ATP-γ-S, 2.5 mM MgCl_2_
Precipitant solution	0.1 M HEPES pH 7.0, 0.2 M MgCl_2_, 10% PEG8K	0.1 M Tris pH 7.2, 10 mM phenol, 24% MPD	0.1 M HEPES pH 7.5, 0.2 M KCl, 35% pentaerythritol propoxylate 5/4
Space group	C 2 2 2_1_	C 2 2 2_1_	P 3_2_ 2 1
Cell parameters			
*a*, *b*, *c* (Å)	88.39, 95.84, 84.39	106.16, 128.00, 131.35	80.68, 80.68, 237.59
*α*, *β*, *γ* (°)	90, 90, 90	90, 90, 90	90, 90, 120
Resolution (Å)	64.97–2.25 (2.30–2.25)*	43.78–1.65 (1.69–1.65)*	34.94–2.53 (2.60–2.53)*
Unique reflexions	16 629 (1186)*	105 524(7381)*	29 335(2139)*
Completeness for range (%)	100 (99.9)*	98.7 (98.5)*	99.9 (99.9)*
Multiplicity	6.5 (6.5)*	4.1 (4.1)*	13.1 (13.6)*
*R*_merge_	0.220 (0.920)*	0.068 (0.608)*	0.061 (0.629)*
*I*/*σ*(*I*)	6.3 (2.1)*	11.0 (2.2)*	23.4 (1.1)*
Wavelength (Å)	0.9686	0.9763	0.97949
Phasing	MR	MR	MR
*R*_work_, *R*_free_ (%)	24.2, 29.8	15.7, 18.1	22.6, 28.4
Number of atoms			
protein, other, solvent	2133, 31, 44	4647, 164, 582	3367, 31, 0
B-factors (Å^2^)			
protein, other, solvent	29.4, 28.6, 20.2	30.8, 21.6, 40.7	94.9, 135.7, —
rmsd bond (Å)	0.015	0.016	0.015
rmsd angle (°)	1.749	1.557	1.846
Ramachandran statistics			
favoured, outliers (%)	96.30, 0.37	97.03, 0.17	90.20, 1.78

* Values in parentheses correspond to the highest resolution shell.

## Results and discussion

### Structure of the LIMK1 kinase domain in complex with ATPγS and CFL1

**Figure 2.**
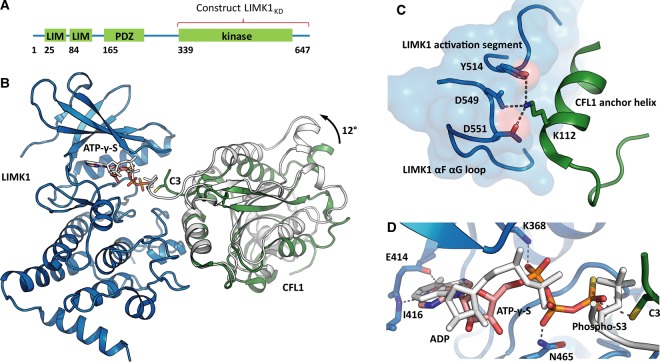
The rock-and-poke mechanism of catalysis. (**A**) LIMK1 is composed of two N-terminal LIM domains, the PDZ domain and the kinase domain. The expression construct LIMK1_KD_ comprised the C-terminus only. (**B**) LIMK1_KD_ (blue) in complex with ATPγS, bound to its physiological substrate CFL1 (green). In CFL1, the serine residue subjected to phosphorylation was mutated to cysteine, in order to trap the enzyme-substrate complex (PDB ID 5L6W). A previously published LIMK1-CFL1 structure (PDB ID 5HVK) showed the complex after the reaction (ADP and Phospho-CFL1 in white, LIMK1 omitted because its conformation was identical with the one shown in blue). The CFL1 binding angles differed by 12°. This rotation resulted in the CFL1 N-terminus poking into the LIMK1 active site (rock-and-poke mechanism). (**C**) The CFL1 anchor helix was firmly attached to the LIMK1 docking interface. The CFL1 residue K112 was co-ordinated by three LIMK1 residues. (**D**) LIMK1 bound its substrate ATPγS in the way typical for kinases. There were two hinge hydrogen bonds to hold the adenine in position. K368 and N465 were adjusting the triphosphate. The CFL1 C3 side chain was oriented for catalysis. For comparison, the enzyme-product complex with ADP and Phospho-CFL1 is again shown in white.

To better characterise substrate recognition by LIMK1, we expressed and purified the recombinant LIMK1 kinase domain (LIMK1_KD_, Figure 2A) from insect cells and full-length CFL1 protein from *E. coli*. Suitable crystals of the LIMK1_KD_–CFL1 complex were obtained using a non-phosphorylatable S3C mutant of CFL1 and the non-reactive ATP analogue ATPγS. The structure of this complex was subsequently solved and refined at 2.5 Å resolution (Figure 2B, PDB ID 5L6W). In the resulting model, LIMK1_KD_ adopted an active kinase conformation with the ATP analogue bound as expected to the kinase hinge region within the ATP-binding pocket. CFL1 was bound as a globular protein made up of a central antiparallel β sheet surrounded by seven α helices. Its N- and C-termini were situated at opposite sides of the molecule. CFL1 interacted with LIMK1 solely via its α5 helix (the anchor helix) which spanned the full width of the LIMK1 kinase domain. This interaction placed the CFL1 N-terminus containing the phosphoacceptor residue in close proximity to the ATP γ phosphate position. Thus, the complex featured the act of phosphoryl transfer and confirmed the recent findings by Hamill et al. [24], who obtained a different crystal form, but the similar model (PDB ID 5HVK) using a catalytic ‘dead' mutant (D460N) of LIMK1. The most striking difference to the previously published model was the orientation of the substrate CFL1 towards LIMK1, which differed by an angle of 12° (Figure 2B). From the analysis of both structure models we deduced a so far undescribed catalytic mechanism that we refer to as the rock-and-poke mechanism.

### A rock-and-poke mechanism for LIMK1 catalytic activity

The structural models rationalised the high specificity of the LIMK1–CFL1 interaction. The LIMK1 interface included parts of the activation loop as well as a large αF–αG loop insertion that displaced the usual αG helix position to specifically accommodate the CFL1 anchor helix (Leu111 to Leu128, Figure 2C). Notably, CFL1 Lys112 was co-ordinated by three LIMK1 residues (Tyr514, Asp549 and Asp551) and was indispensable for substrate recognition, as discussed below (Figure 2C). These interactions were supported by additional hydrophobic contacts including CFL1 Met115. While CFL1 was firmly attached to LIMK1, the 12° rotation between the new structure and that of Hamill et al. suggested that the CFL1 molecule could rock back and forth around the anchor helix resulting in the CFL1 phosphoacceptor poking in and out towards the ATP γ phosphate to establish a constructive orientation for phosphoryl transfer (Figure 2B,D). This rock-and-poke mechanism is different from the conventional catalytic mechanism of kinases. Usually, the phosphoacceptor residue is embedded into an unstructured linear motif or a flexible loop that transiently forms β-sheet-like hydrogen bonding to the kinase activation loop for phosphoryl transfer [33]. In this regard, LIMK1 is unusual in that it catalyses tip phosphorylation, rather than the common loop phosphorylation.

### LIMK1 is a dual-specificity kinase

**Figure 3.**
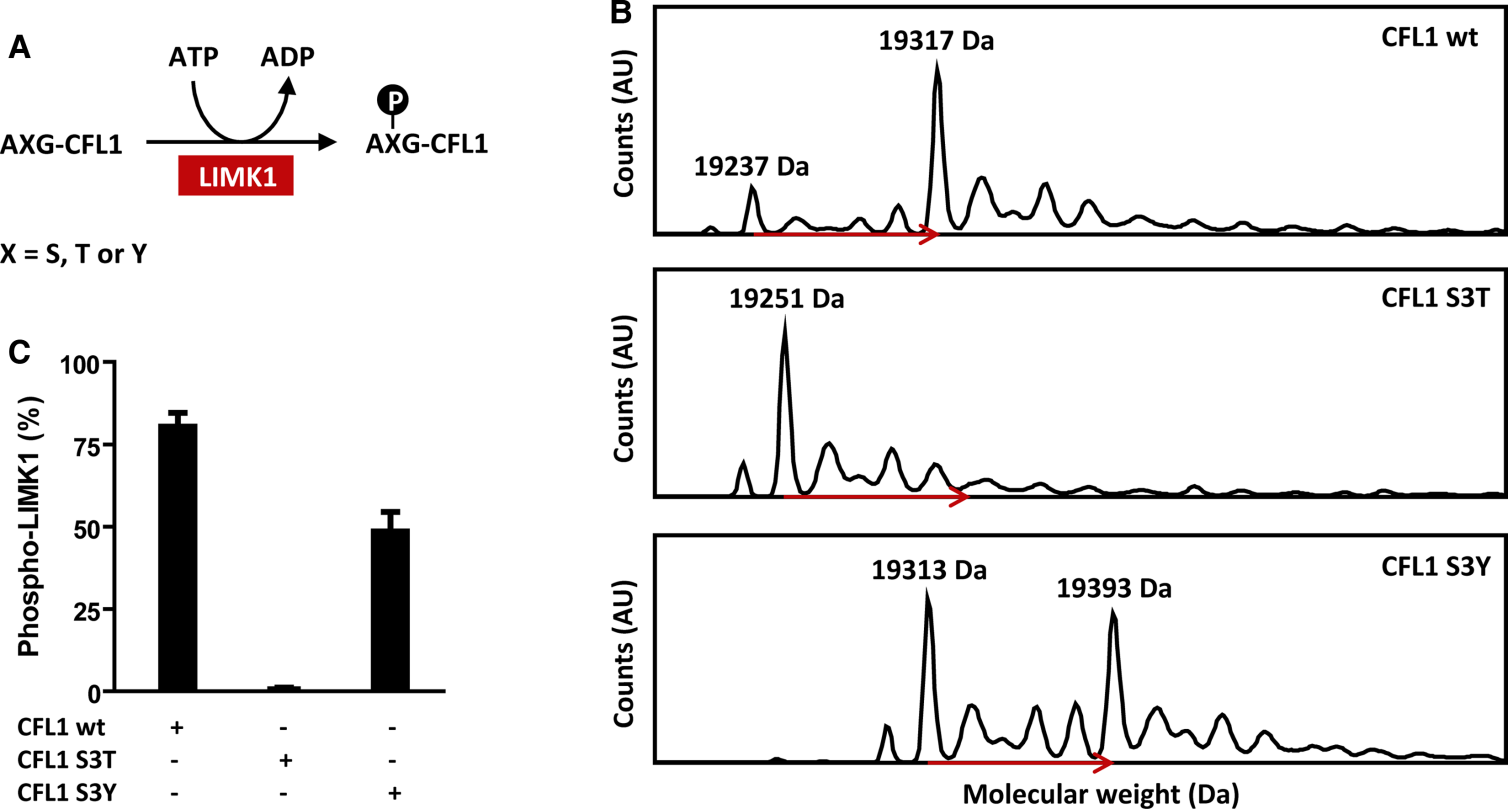
LIMK1 is a dual-specificity kinase. (**A**) CFL1 variants were tested as LIMK1_KD_ substrates. AXG is the amino acid sequence of the CFL1 N-terminus. The X stands for S (CFL1 wt), T and Y, respectively. Please note M1 was cleaved during expression in bacteria. (**B**) Deconvoluted mass spectra of the reaction mixtures containing either CFL1 wt, S3T or S3Y. Phosphorylation induced an 80 Da shift to the CFL1 mass as indicated by the red arrows. (**C**) Comparison of the CFL1 phosphorylation grades. Both CFL1 wt and S3Y were good LIMK1 substrates, which makes LIMK1 a dual-specificity kinase. CFL1 S3T was not phosphorylated by LIMK1. The experiment was performed in triplicate.

Conventionally, the depth of the phosphoacceptor residue pocket determines if a protein kinase is a Ser/Thr or Tyr-specific enzyme [33]. The rock-and-poke mechanism would allow for variable phosphoacceptor positions potentially explaining previous reports that LIMK1 exhibited dual substrate specificity [34]. To investigate this possibility, we produced CFL1 substrate variants containing either a S3T or S3Y substitution in addition to the wild-type protein. LIMK1, ATP and respective CFL1 variants were then incubated to allow for phosphorylation (Figure 3A), and the mixtures analysed by mass spectrometry (Figure 3B). Under the chosen reaction conditions, 80% of wild-type CFL1 (Ser3) and 50% of the CFL1 S3Y mutant were phosphorylated confirming LIMK1 as a dual-specificity kinase. Surprisingly, no phosphorylation was detected on the CFL1 S3T mutant (Figure 3C). Large scale kinase studies have indicated that large hydrophobic residues (Leu, Phe, or Met) at the activation loop DFG + 1 position confer kinases with selectivity for Ser over Thr residues [35]. Leu481 occupies this position in LIMK1 and presumably creates steric hindrance for the binding of the branched Thr residue. In this respect, the rock-and-poke mechanism conforms to the precedents of the more conventional loop phosphorylation.

### LIMK1 is targeted by clinically tested kinase inhibitors

**Figure 4.**
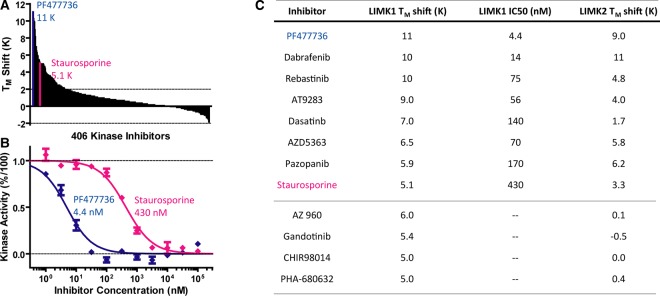
Identification and confirmation of LIMK1 inhibitors. (**A**) The effect of 407 conventional kinase inhibitors on the LIMK1 thermal stability was determined in DSF assay. PF-477736 induced the highest shift with 11 K, staurosporine stabilised LIMK1 by 5.1 K. (**B**) The inhibitory potential of the best DSF hits was probed in the RapidFire kinase activity assay. The dose-response curves for PF-477736 and staurosporine are plotted in blue and red, respectively. (**C**) The results obtained from DSF and RapidFire assays were in excellent agreement. Several inhibitors stabilised LIMK1, but not LIMK2. These inhibitors were isoform-specific (see also Supplementary Table S1).

The conservation of the kinase domain ATP-binding pocket presents challenges for the design of selective inhibitors, but also provides opportunities for drug repurposing. Indeed, the vast majority of clinically tested compounds are multi-targeted kinase inhibitors. We utilised differential scanning fluorimetry (DSF) [28] as an initial screen to test LIMK1 for binding to a collection of these clinical molecules available commercially through the Selleckchem L1200 compound library. Hits in this assay stabilise the protein's melting temperature (*T*_M_) proportionally to the negative log of the IC_50_ value (pIC_50_). In the presence of buffer alone LIMK1_KD_ displayed a *T*_M_ value of 49°C. A number of the tested compounds stabilised LIMK1_KD_ substantially, with PF-477736 (11 K) and dabrafenib (10 K) inducing the highest *T*_M_ shifts (Figure 4A). For comparison, the library was similarly screened for its potential to stabilise LIMK2_KD_. As expected, the best LIMK1_KD_ binding compounds also stabilised LIMK2_KD_ (Figure 4C). However, several inhibitors displayed interesting isoform specificity, namely AZ 960 (LIMK1_KD_ 6.0 K, LIMK2_KD_ 0.1 K) and gandotinib (5.4 K, −0.5 K). These compounds provide proof of principle that LIMK1 can be targeted selectively over LIMK2 as well as potential chemical starting points for the development of LIMK1-specific inhibitors. All *T*_M_ shift values are listed in Supplementary Table S1.

### A RapidFire mass spectrometry assay for IC_50_ determination

The DSF assay offers a robust format for screening with a high dynamic range, but confirmation in an orthogonal *in vitro* kinase assay is desirable for hit validation as well as to compare results across different kinases. To establish suitable assay conditions, we initially tested a set of 20 commercial peptides that are known to be phosphorylated by a wide range of kinases as potential substrates for LIMK1 (Supplementary Table S2). No activity towards these peptides was observed consistent with the divergent substrate-binding pocket of LIMK1 and its unusual rock-and-poke mechanism. We, therefore, switched to recombinant CFL1 as a known substrate and employed a mass spectrometry assay to monitor LIMK1_KD_-dependent phosphorylation of CFL1 Ser3. An ATP concentration of 800 µM was adopted for IC_50_ measurements corresponding to the kinase's apparent *K*_M_ value for ATP. Analysis of the reaction mixtures by mass spectrometry revealed baseline-separated peaks for the non-phosphorylated and phosphorylated (+80 Da) CFL1 proteins that enabled clear quantification of the reaction product. Phosphomapping of the reaction product confirmed that LIMK1_KD_ phosphorylated CFL1 at Ser3 only. To profile the top hits from the DSF screening, we took advantage of a mass spectrometer equipped with a RapidFire sample robot allowing for small reaction volumes (50 µl per reaction) and high performance (90 min per 384 samples) as well as Agilent software for automated peak integration. Example inhibition curves are shown in Figure 4B and reveal IC_50_ values for LIMK1 inhibition ranging from 4.4 nM (PF-477736) to 430 nM (staurosporine). Overall, all the identified hits were confirmed as LIMK1 inhibitors with excellent agreement between the ranking of compounds by the DSF and RapidFire assays (Figure 4C).

### Exploring the conformational space for inhibitor interaction with LIMK1_KD_

**Figure 5.**
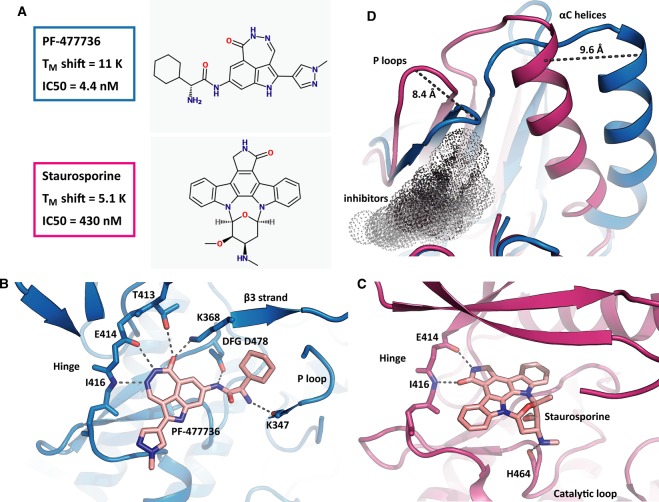
Examples of inhibitors bound to LIMK1. (**A**) Binding properties and chemical structures of PF-477736 (blue) and staurosporine (red). (**B**) The LIMK1-PF-477736 complex (PDB ID 5NXC). Beside the hinge interactions including the gatekeeper residue T413, the inhibitor bridged the DFG motif, the β3 sheet and the P loop. Parts of the model were removed for clarity. (**C**) The LIMK1-staurosporine complex (PDB ID 3S95). In addition to the two salt bridges to the LIMK1 hinge, there is only the interaction to H464 prior to the β7 sheet. This explains the low affinity of the compound. (**D**) Overlay of the PF-477736 (blue) and staurosporine (red) structures, as seen from the active site in the direction of the N-lobe. Staurosporine bound LIMK1 in an active-like conformation with intact K-E salt bridge, while PF-477736 binding induced an inactive conformation, characterised by a 9.6 Å outwards shift of αC helix, with no K-E salt bridge formed. The dark cloud represents the inhibitor binding site.

While several LIMK1 inhibitors are described in the literature [17,20], the absence of structural models for their interaction limits the prospects of rationally optimising their binding potency and selectivity. To address this need we performed further co-crystallisation trials with our recombinant LIMK1_KD_ protein and solved the structures of LIMK1 in complexes with the top screening hit PF-477736 (PDB ID 5NXC) as well as staurosporine (PDB ID 3S95) (Figure 5A). PF-477736 was developed as a checkpoint kinase-1 (Chk1) inhibitor and induces a ‘synthetic lethal’ effect in DNA-damaged cancer cells by disrupting the S-G_2_ checkpoint [36]. The co-structure with LIMK1 showed an ATP-competitive binding mode with two hydrogen bonds between the diazapenyl moiety nitrogens and the kinase hinge region, as well as a further bifurcated hydrogen bond between the carbonyl substituent and the side chains of the gatekeeper residue Thr413 and catalytic lysine (Lys368, Figure 5B). Two additional hydrogen bonds were formed between the amide and primary amines of PF-477736 with the DFG motif Asp478 and P-loop Lys347, respectively (Figure 5B). The natural product staurosporine is a widely studied promiscuous kinase inhibitor [37]. Its binding mode exhibited similarly extended hydrophobic contact, but fewer electrostatic interactions, perhaps reflecting its lower affinity for LIMK1 (Figure 5A). As typically observed for this compound, staurosporine formed two hydrogen bonds with the kinase hinge as well as an interaction with the backbone carbonyl of the catalytic loop residue His464 (Figure 5C).

Comparison of the two inhibitor-bound structures revealed a significant 10 Å shift in the position of the αC helix as well as an 8 Å shift in the P-loop (Figure 5D). The LIMK1 complex with the type I inhibitor staurosporine showed a DFG-in αC-in conformation with an intact catalytic salt bridge between the β3 lysine and the αC glutamate that is characteristic for an active kinase. Both the C-spine and the R-spine of the kinase were intact, further confirming the active conformation of the catalytic domain. No crystal structures were previously solved using PF-477736 despite this compound entering clinical trials. However, PF-477736 was formerly modelled into the CHK1 kinase structure as a classical type I inhibitor binding to an active αC-in conformation (template PDB ID 4FT5, [38]). Our structure revealed a distinct flipped conformation of PF-477736 that switched the hydrogen bond interaction of the diazapenyl moiety carbonyl from the hinge +3 position (Chk1 model, [38]) to the gatekeeper and catalytic lysine (LIMK1, PDB ID 5NXC). Consequently, the catalytic salt bridge was broken in the LIMK1 co-structure and a DFG-in αC-out conformation was observed (Figure 5D). Interestingly, the LIMK1 R-spine was broken due to the outward shift of the αC. The Met388 site chain was removed from the R-spine and the resulting space filled with the Phe399 side chain. These major rearrangements were accompanied by a closed conformation of the P-loop which packed closely with the phenyl ring of PF-477736 (Figure 5B). The weaker interaction of staurosporine was associated with a more open conformation of the P-loop resulting in less packing interactions and higher B factors.

### Strategies to enhance LIMK1 kinase activity for enzymology studies

**Figure 6.**
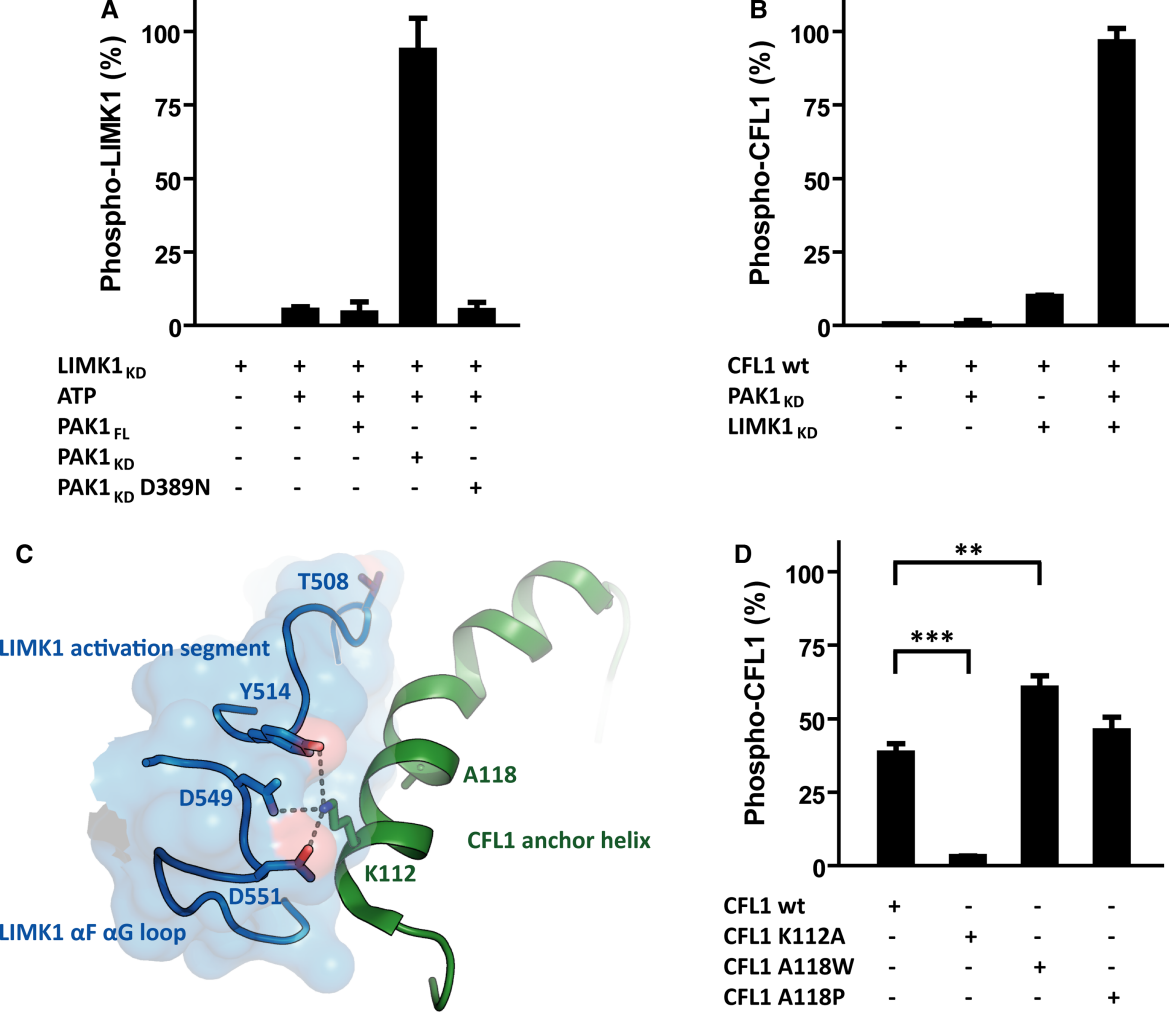
Modulating the LIMK1 enzyme and the CFL1 substrate properties. (**A**) Recombinant PAK1_KD_ phosphorylated recombinant LIMK1_KD_, while PAK1_FL_ and PAK1_KD_ D389N were not active. Please note the basal LIMK1 autophosphorylation. (**B**) Phosphorylation increased LIMK1 kinase activity by more than factor 10. PAK1_KD_ did not phosphorylate CFL1 at all. (**C**) Binding of the CFL1 anchor helix to the LIMK1 docking interface. Residues important for binding and residues subjected to mutagenesis are highlighted. (**D**) Impact of amino acid exchanges in the CFL1 anchor helix on the CFL1 substrate properties. CFL1 K112 was indispensable for substrate recognition. The introduction of tryptophan in position 118 made CFL1 an even better LIMK1 substrate. Breaking the anchor helix in position 118 did not significantly alter substrate recognition. All experiments were performed in triplicate.

LIMK1 can be phosphorylated under physiological conditions by the kinase PAK1 (Figure 1A) [39]. Recombinant LIMK1_KD_ purified from insect cells was not phosphorylated as determined by mass spectrometry (Figure 6A). We incubated this protein with different constructs of PAK1 *in vitro* to assess their impact on LIMK1 phosphorylation state and catalytic activity. All phosphorylation experiments were performed as three independent repeats. The isolated PAK1 kinase domain demonstrated robust phosphorylation of LIMK1_KD_, whereas no significant phosphorylation of LIMK1_KD_ was observed using the kinase-dead variant PAK1_KD_ D389N or using full-length PAK1 containing its autoinhibitory domain (Figure 6A). Phosphomapping confirmed that the single phosphorylation occurred at LIMK1 Thr508 located within the kinase domain activation loop (data not shown). Of note, LIMK1_KD_ alone also showed a low level of autophosphorylation when mixed with ATP and MgCl_2_ (Figure 6A), although the physiological relevance of this result remains unclear. Importantly, phosphorylation at Thr508 increased the LIMK1_KD_ activity towards its substrate CFL1 by more than 10-fold (Figure 6B), providing an option to improve the sensitivity of the RapidFire activity assay. Thr508 was situated in the LIMK1 activation segment. Upon phosphorylation, it interacted with Arg483 [24], likely stabilising the interface recognised by the CFL1 anchor helix.

We also explored whether mutations in the CFL1 anchor helix could lead to enhancements in the LIMK1_KD_ activity. The mutant CFL1 A118W was engineered to increase the potential size of the protein–protein interaction surface. Most interestingly, this CFL1 variant proved to be a more favourable substrate for LIMK1_KD_ than the wild-type protein (Figure 6C). This potentially reflects π stacking interactions of the tryptophan with the loop C-terminal to the LIMK1 APE motif. Perhaps surprisingly, this CFL1 position appeared rather tolerant to substitution. The introduction of a proline in the mutant CFL1 A118P would be expected to break the anchor helix. However, this mutation did not alter the CFL1 substrate properties (Figure 6C). This result suggested that the key CFL1 residues for LIMK1 interaction occur at the N-terminal end of the anchor helix. Finally, as a negative control we introduced the mutation CFL1 K112A which breaks ion pair interactions with LIMK1 Asp549 and Asp551. As anticipated from the structural model, CFL1 K112A was only poorly phosphorylated by LIMK1 (Figure 6C), highlighting the critical role of this residue in binding.

## Discussion

When analysing the co-ordinates of our LIMK1–ATPγS–CFL1 complex (PDB ID 5L6W), the distribution of B factors was remarkable. While the B factors of residues in the LIMK1 kinase core were relatively low, the B factors in the entire CFL1 molecule were high. This indicated a lack of CFL1 rigidity due to the transient, dynamic nature of the enzyme-substrate interaction. We then compared our structure model to the recently published LIMK1–ADP–Phospho-CFL1 model (PDB ID 5HVK) [24]. The difference in the CFL1 binding angle was intriguing, further supporting the idea of a loosely bound CFL1 subunit. The structural models resulted from different crystal forms where crystal packing differences have the potential to influence the CFL1 binding angle. However, we regard the two structural models as two snapshots of how the enzyme-substrate complex might look in solution at a certain point in the catalytic process. From these two snapshots, the rock-and-poke mechanism suggested herein was deduced. It may be interesting in future work to explore this mechanism by molecular dynamics simulation.

Our LIMK1–ATPγS–CFL1 crystals were characterized by an unusually high water content of ∼70%. CFL1, in particular, was not stabilised by many crystal contacts and in the crystal lattice rather formed a bridge between two LIMK1 molecules. Therefore, the CFL1 loop regions retained some flexibility in the crystal. Several residues in the CFL1 loops were Ramachandran outliers despite extensive model building. However, the unusual conformations of CFL1 Lys44, Ile131 and Ala157 fall at positions far from the binding interface and are therefore technical rather than key structural features.

In the eukaryotic cell, CFL1 is found either in its free form or in its actin filament-bound form. Importantly, actin binding is mediated by the CFL1 anchor helix — the same helix that is indispensable for LIMK1 substrate recognition. Consequently, LIMK1 can only phosphorylate CFL1 in its free form. The assays and the structure models presented herein will hopefully facilitate the development of potent and specific chemical probes for LIMK1 functional studies. For example, would LIMK1 inhibition alone be sufficient to reduce the phosphorylation state of the CFL1 pool, or would this still differ according to cell type and developmental stage due to the occurrence of LIMK2, TESK1/2 and the phosphatase slingshot? It also remains unclear whether LIMK1 can phosphorylate other substrate proteins in addition to ADFs. A greater understanding of these questions would help to inform on the safety and efficacy of potential LIMK1-specific therapeutics as a future treatment for diseases such as FXS and ALS.

## Conclusions

The physiological role of LIMK1 is to phosphorylate the very N-terminus (Ser3) of its substrate proteins. Therefore, LIMK1 substrate recognition and catalytic mechanism differ from what is common in conventional kinases. Substrates such as CFL1 bind specifically to the LIMK1 docking interface via their anchor helices that are <100 residues distant from the phosphoacceptor residue. All LIMK inhibitors published to date target the ATP-binding site. Small molecules mimicking the CFL1 anchor helix might represent a worthwhile alternative approach to inhibit LIMK1 kinase activity. The requirement to phosphorylate the tip of a polypeptide chain is met by the rock-and-poke mechanism of catalysis. As a consequence, LIMK1 is a dual-specificity kinase that phosphorylates both Ser and Tyr residues. However, phosphorylation of the branched Thr residue was not observed. The lessons from enzymology helped to design and to improve a RapidFire mass spectrometry-based activity assay that combined the use of the physiological substrate CFL1 with keeping the throughput at least medium (∼250 data points per hour). With our limited inhibitor screen a set of LIMK1 inhibitors was identified, some of which notably were isoform specific (no binding to LIMK2). The first crystal structures of LIMK1 in complex with inhibitors revealed the plasticity of the kinase domain. Depending on the bound inhibitor, the P loop and the αC helix shifted substantially. This deepened understanding of LIMK1 enzymology and structure will hopefully facilitate future inhibitor screening and medicinal chemistry efforts.

## Abbreviations

ADF: actin-depolymerising factor
ALS: amyotrophic lateral sclerosis
CFL1: cofilin-1
DSF: dynamic scanning fluorimetry
FMR1: Fragile X mental retardation 1
FXS: fragile X syndrome
KD: kinase domain
LIMK1: LIM domain kinase 1
MGC: mammalian gene collection
ROCK1: Rho-associated protein kinase 1
SPE: solid-phase extraction
