# Supplementary material for: Lessons from LIMK1 enzymology and their impact on inhibitor design
Source: Biochem J. 2019 Nov 5;476(21):3197–209. doi: 10.1042/BCJ20190517 (PMC6835155; doi:10.1042/BCJ20190517)
Supplement: Supplementary Tables S1-S2 [file BCJ-476-3197-s1.pdf]

Table S1 TM shifts

Plate	Well	Compound	T_M Shift LIMK1 (K)	T_M Shift LIMK2 (K)	LIMK1 specific
L1200-4	F03	PF-477736	11.1	9.0	
L1200-3	D11	Dabrafenib (GSK2118436)	10.3	11.1	
L1200-3	F03	DCC-2036 (Rebastinib)	10.0	4.8	
L-1200-1	A10	AT9283	9.0	4.0	
L1200-2	C02	JNJ-7706621	8.2	5.8	
L1200-4	F02	IKK-16 (IKK Inhibitor VII)	7.0	2.1	
L-1200-1	E2	Dasatinib	7.0	1.7	
L1200-4	A11	AZD5363	6.5	5.8	
		Momelotinib	6.3	4.2	
L1200-2	C11	AZ 960	6.0	0.1	
L1200-4	D04	Pazopanib	5.9	6.2	
L-1200-1	F3	Pazopanib HCl (GW786034 HCl)	5.5	6.9	
		Tamatinib	5.5	3.0	
		Gandotinib	5.4	-0.5	
L1200-2	G08	TWS119	5.2	4.1	
		Staurosporine	5.1	3.3	
		Lestaurtinib	5.0	2.0	
L1200-2	G05	Ponatinib (AP24534)	5.0	4.6	
L1200-3	D08	CHIR-98014	5.0	0.0	
L-1200-1	C8	Danusertib (PHA-739358)	5.0	1.0	
L1200-2	H09	LY2784544	5.0	1.1	
L1200-2	C04	PHA-680632	5.0	0.4	
L1200-2	G02	BX-795	5.0	4.7	
L1200-2	B04	Aurora A Inhibitor I	4.9	3.7	
		AZD1480	4.7	0.6	
L1200-2	D05	AEE788 (NVP-AEE788)	4.7	4.5	
L1200-2	B01	ENMD-2076	4.6	6.0	
L1200-3	H02	AZD5438	4.4	1.7	
L1200-4	A06	XL019	4.0	1.6	
L-1200-1	F5	Crizotinib (PF-02341066)	3.9	1.4	
L1200-2	G07	BMS-777607	3.8	-0.2	
L1200-4	A08	AVL-292	3.8	3.1	
L1200-3	F06	TAK-901	3.7	0.3	
		ASP3026	3.7	1.4	
L-1200-1	E11	CYC116	3.7	1.6	
L1200-3	A03	PP121	3.6	2.7	
L1200-2	H02	BX-912	3.6	2.8	
		AZ23	3.6	0.8	
L1200-2	E03	MGCD-265	3.6	1.1	
L1200-4	A03	Tyrphostin 9	3.5	6.3	
L1200-4	B05	AP26113	3.4	0.1	
L-1200-1	G4	VX-680 (Tozasertib, MK-0457)	3.4	0.0	
L1200-3	C08	CCT137690	3.4	3.5	
L1200-2	D01	PIK-75	3.3	2.4	
		Fedratinib	3.2	1.4	
		Ralimetinib	3.2	3.1	
L1200-2	A01	WZ8040	3.1	5.2	
L1200-4	B04	CHIR-99021 (CT99021) HCl	3.1	-0.3	
L1200-4	A02	Golvatinib (E7050)	3.0	1.0	
		PF 431396	2.9	0.2	
		(5Z)-7-Oxozeaenol	2.8	0.7	
L1200-4	D01	TPCA-1	2.8	3.6	
L1200-2	F03	Rigosertib (ON-01910)	2.7	1.6	
L1200-2	E06	Hesperadin	2.7	1.2	
L-1200-1	E9	Cabozantinib (XL184, BMS-907351)	2.5	0.6	
L-1200-1	F10	PLX-4720	2.5	3.8	
L1200-2	C05	HMN-214	2.5	1.0	
L1200-4	A07	AZD3463	2.5	3.1	
L1200-2	A02	AG-1024	2.5	4.3	
		Bafetinib	2.5	1.1	
		LY2874455	2.5	0.1	
L1200-3	A05	Ibrutinib (PCI-32765)	2.4	0.7	

L1200-2	A06	CCT129202	2.4	4.6
L1200-3	H05	TG101209	2.4	3.1
		Ipatasertib	2.3	2.3
L-1200-1	D8	TAE684 (NVP-TAE684)	2.3	0.8
L1200-4	G01	BI-D1870	2.2	3.9
L1200-3	A10	Dovitinib (TKI-258) Dilactic Acid	2.2	3.8
L1200-3	B03	OSI-027	2.2	0.2
L1200-3	D05	CHIR-124	2.2	-1.7
		Pictilisib	2.2	0.1
L1200-2	H05	LY2228820	2.1	4.4
L1200-2	E10	R406	2.0	1.2
L1200-4	D06	PP1	2.0	0.0
L1200-3	G07	TG101348 (SAR302503)	2.0	0.7
		Linifanib	2.0	5.0
L-1200-1	B1	Axitinib	2.0	-0.7
L1200-3	A09	HER2-Inhibitor-1	2.0	0.2
L1200-2	E11	PP242	2.0	3.7
L1200-4	E05	PP2	2.0	2.4
		Orantinib	1.9	1.0
L1200-2	E07	KRN 633	1.9	1.4
		Alvocidib	1.9	0.5
L-1200-1	F8	Foretinib (GSK1363089)	1.9	-0.4
L-1200-1	D11	WZ3146	1.9	1.5
		Sunitinib	1.8	-0.1
L1200-3	F07	MK-8776 (SCH 900776)	1.8	2.2
L1200-2	F08	H 89 2HCl	1.8	3.9
		BMS-911543	1.8	0.3
L1200-3	D06	KX2-391	1.7	-0.3
L1200-2	H04	TSU-68 (SU6668, Orantinib)	1.7	2.8
L1200-2	F10	SGI-1776 free base	1.7	1.4
L-1200-1	B4	Sorafenib Tosylate	1.7	1.5
L1200-2	F06	BIX 02188	1.7	-0.1
L1200-3	D07	SB415286	1.7	0.9
L1200-2	E09	KW-2449	1.7	0.8
		Baricitinib	1.6	1.0
		PF-04708671	1.6	1.7
L1200-2	H03	Ruxolitinib (INCB018424)	1.6	2.6
		Apitolisib	1.6	0.6
L1200-4	A09	CNX-774	1.6	1.2
L-1200-1	B2	Pacritinib (SB1518)	1.6	1.5
L1200-4	F08	Ro 31-8220 Mesylate	1.6	1.2
L-1200-1	C4	Sunitinib Malate	1.6	0.5
		PF-670462	1.5	-0.2
L1200-2	G10	BMS-794833	1.5	2.0
L1200-2	F02	Vemurafenib (PLX4032, RG7204)	1.5	6.0
L1200-3	E05	NVP-BSK805 2HCl	1.5	0.2
L1200-3	B10	MK-5108 (VX-689)	1.5	0.9
L1200-4	C02	WHI-P154	1.5	-0.2
L1200-3	C07	AG-1478 (Tyrphostin AG-1478)	1.4	-0.2
		GSK650394	1.4	-0.2
L-1200-1	E6	SU11274	1.4	-0.2
L1200-3	F11	INK 128 (MLN0128)	1.4	0.3
L1200-3	B02	Indirubin	1.4	2.6
L1200-3	H04	Flavopiridol HCl	1.4	1.4
L1200-4	D08	RKI-1447	1.4	0.8
L1200-4	D02	TG100713	1.4	0.7
L1200-3	A06	GDC-0980 (RG7422)	1.4	0.6
L1200-4	A10	AR-A014418	1.4	1.0
L1200-4	B11	TCS 359	1.3	1.1
L1200-3	A07	PH-797804	1.3	0.1
L1200-4	C09	TAK-632	1.3	3.2
		Nintedanib	1.3	0.3
L1200-3	F08	AMG-458	1.3	-0.8

L-1200-1	G5	PHA-665752	1.3	-0.4
L1200-3	H07	GSK1070916	1.3	-0.1
		PF-4800567	1.3	-0.8
L-1200-1	B6	SB203580	1.3	3.3
L1200-2	C03	TG100-115	1.3	2.4
L1200-2	E08	Tie2 kinase inhibitor	1.3	1.4
L1200-3	C06	CH5132799	1.3	0.1
L-1200-1	C1	Saracatinib (AZD0530)	1.2	0.8
L1200-2	C06	AT7519	1.2	1.3
		MK-1775	1.2	0.0
L1200-2	H06	AZD7762	1.2	1.1
L1200-3	A08	PHA-767491	1.2	0.4
L1200-3	B04	GSK2126458 (GSK458)	1.2	0.9
L1200-2	F11	CYT387	1.2	0.8
L1200-2	D03	GSK1059615	1.2	2.6
		UCN-01	1.2	0.7
L1200-4	B01	TAE226 (NVP-TAE226)	1.1	3.3
L1200-2	D02	PD173074	1.1	1.2
L1200-3	F04	PF-00562271	1.1	0.3
L1200-2	E04	Thiazovivin	1.1	1.0
L1200-2	F09	RAF265 (CHIR-265)	1.1	5.1
L1200-2	E01	Tivozanib (AV-951)	1.1	1.5
L1200-2	D10	GSK461364	1.1	0.8
L-1200-1	A5	Enzastaurin (LY317615)	1.0	1.6
L1200-2	B06	SAR245409 (XL765)	1.0	6.6
L1200-2	A07	R406 (free base)	1.0	1.2
		GSK269962	1.0	0.1
		Ruboxistaurin	1.0	0.4
L1200-2	F05	PIK-93	1.0	1.7
L1200-2	F04	SP600125	1.0	2.1
L1200-3	E07	Crenolanib (CP-868596)	1.0	0.5
L1200-3	C03	Fostamatinib (R788)	1.0	0.5
L1200-2	A09	PF-573228	1.0	0.7
L1200-2	E05	PHA-793887	1.0	4.1
L1200-3	C11	CEP-33779	1.0	1.5
L1200-3	C02	Quercetin	1.0	3.9
L1200-4	E06	MK-8745	1.0	-0.3
L-1200-1	F11	WZ4002	1.0	1.8
L1200-3	B09	Varlitinib	0.9	0.8
L-1200-1	C2	Canertinib (CI-1033)	0.9	0.5
L1200-2	C07	AZD8055	0.9	0.8
L1200-2	C10	AZD8931 (Sapitinib)	0.9	0.6
L1200-3	B08	PF-04691502	0.9	0.6
L1200-3	A02	Honokiol	0.9	1.7
L1200-2	B07	CP-673451	0.9	1.9
L1200-3	B06	A-769662	0.9	2.4
L1200-3	D02	Chrysophanic Acid	0.9	1.3
L1200-3	B07	Dacomitinib (PF299804, PF299)	0.9	0.2
L1200-4	E03	S-Ruxolitinib (INCB018424)	0.9	-0.7
L1200-2	F07	AT7867	0.9	1.4
L1200-4	G07	LY2835219	0.9	0.3
L1200-3	B01	PIK-294	0.8	1.2
L1200-4	F10	VE-821	0.8	1.5
L-1200-1	B7	GSK1904529A	0.8	0.7
L1200-4	B07	NU6027	0.8	-0.9
L1200-3	C10	MK-2461	0.8	0.3
L1200-4	C06	AZ20	0.8	-0.8
L-1200-1	F6	Brivanib (BMS-540215)	0.8	0.3
L-1200-1	D4	Tandutinib (MLN518)	0.8	0.0
L1200-2	C08	Fasudil (HA-1077) HCl	0.8	0.9
L1200-2	B05	Pimasertib (AS-703026)	0.8	2.1
L1200-3	D04	A-674563	0.8	0.2
L1200-3	E02	Imatinib (STI571)	0.8	1.0

L1200-2	C01	CUDC-101	0.8	1.6
		Linsitinib	0.8	0.9
L1200-3	H06	ZM 336372	0.7	0.2
L-1200-1	B10	Brivanib Alaninate (BMS-582664)	0.7	0.0
L-1200-1	F1	P276-00	0.7	1.3
L1200-2	G01	OSI-930	0.7	6.2
L1200-4	D07	CGK 733	0.7	4.2
L1200-2	B09	BMS-265246	0.7	1.4
		Sonolisib	0.7	0.6
		BMS-599626	0.7	-0.4
L1200-2	A10	BGJ398 (NVP-BGJ398)	0.7	0.5
L1200-3	C05	CAY10505	0.7	2.3
L-1200-1	C10	AG-490 (Tyrphostin B42)	0.7	0.9
L-1200-1	C6	SB202190 (FHPI)	0.7	0.2
L-1200-1	B5	AC480 (BMS-599626)	0.7	0.9
L-1200-1	A6	SB216763	0.7	1.3
L-1200-1	E5	SL-327	0.7	0.7
L1200-2	H08	Acadesine	0.7	2.1
		PF-03758309	0.7	-0.4
L1200-2	B08	BS-181 HCl	0.7	1.7
L-1200-1	A11	Lenvatinib (E7080)	0.7	0.1
L1200-2	G04	AZD6482	0.7	1.8
		AZD1152-HQPA	0.6	0.7
L1200-2	D07	PHT-427	0.6	1.6
L1200-2	B11	PIK-293	0.6	0.9
L1200-3	H10	Sotrastaurin	0.6	1.2
L1200-2	H07	PD318088	0.6	2.8
L1200-2	D04	VX-745	0.6	1.3
L1200-2	D11	Mubritinib (TAK 165)	0.6	-0.4
L-1200-1	C3	Motesanib Diphosphate (AMG-706)	0.6	0.5
L1200-4	A05	VX-702	0.6	-0.6
		Buparlisib	0.6	0.0
L1200-2	F01	YM201636	0.6	3.8
		GSK2334470	0.5	1.1
		Idelalisib	0.5	-0.8
L-1200-1	B9	Palbociclib (PD-0332991) HCl	0.5	0.2
L1200-4	E07	AZD1080	0.5	2.3
L1200-2	A08	KU-60019	0.5	0.6
L1200-2	A05	GSK429286A	0.5	1.4
L1200-3	D01	Volasertib (BI 6727)	0.5	0.3
L1200-3	C04	WYE-125132 (WYE-132)	0.5	0.9
L-1200-1	D3	Nilotinib (AMN-107)	0.5	0.4
L1200-4	C07	TIC10	0.5	-1.2
L1200-3	A04	NU7441 (KU-57788)	0.5	-0.9
L1200-4	G03	Go 6983	0.5	1.8
L1200-2	D08	BIRB 796 (Doramapimod)	0.5	0.9
L1200-3	G06	AMG-900	0.5	-2.1
L1200-4	C04	TAK-715	0.4	2.6
L1200-4	C03	ZM 306416	0.4	1.8
L-1200-1	D6	MK-2206 2HCl	0.4	-1.1
L-1200-1	B11	CP-724714	0.4	-0.8
L1200-2	B10	AST-1306	0.4	0.1
L1200-4	D05	MEK162 (ARRY-162, ARRY-438162)	0.4	2.1
L1200-4	B06	PD168393	0.4	2.0
L1200-4	E09	WZ4003	0.4	-0.8
L1200-3	E06	GSK1838705A	0.4	1.0
		AZ3146	0.4	0.0
L1200-3	G09	3-Methyladenine	0.4	0.5
L1200-4	H11	BMS-345541	0.4	2.8
L1200-4	B09	CO-1686 (AVL-301)	0.4	-0.4
L-1200-1	C5	Masitinib (AB1010)	0.4	0.0
L1200-3	G03	CCT128930	0.4	1.0
L1200-2	G09	PF-4708671	0.4	3.5

		SCH772984	0.3	0.1
		Galunisertib	0.3	-0.5
L1200-2	H01	KU-0063794	0.3	2.0
L1200-4	F07	10058-F4	0.3	1.7
L-1200-1	C7	PF-04217903	0.3	-0.1
		Dactolisib	0.3	-0.3
L1200-3	B05	AS-604850	0.3	2.2
L1200-3	E08	AZ 628	0.3	1.2
L1200-3	D09	CUDC-907	0.3	-0.1
		EMD1214063	0.3	0.3
L-1200-1	G1	Afatinib (BIBW2992)	0.3	0.3
L1200-2	C09	AZD8330	0.3	0.2
L-1200-1	G9	BMS-754807	0.2	-0.6
L1200-2	D09	Neratinib (HKI-272)	0.2	1.0
L-1200-1	B3	Lapatinib (GW-572016) Ditosylate	0.2	1.8
L1200-4	F06	LDK378	0.2	-0.3
L-1200-1	H6	OSI-906 (Linsitinib)	0.2	0.7
		Voxtalisib	0.2	0.4
L1200-4	C10	PQ 401	0.2	-0.9
L1200-2	H11	Apatinib	0.2	3.8
L-1200-1	E10	Barasertib (AZD1152-HQPA)	0.2	-0.2
L-1200-1	G3	PD0325901	0.2	1.5
L-1200-1	G6	NVP-ADW742	0.2	0.1
L1200-3	H08	Milciclib (PHA-848125)	0.2	0.6
L1200-3	D10	AZD2014	0.2	-0.3
L1200-3	C09	Wortmannin	0.2	0.1
L1200-4	E08	BIO	0.2	2.2
L1200-4	D10	ZM 39923 HCl	0.2	-1.4
L1200-2	E02	WYE-354	0.2	4.1
L1200-3	D03	LY2603618	0.1	0.7
L1200-3	H11	Tyrphostin AG 879	0.1	1.2
L-1200-1	F2	Ridaforolimus (Deforolimus, MK-8669)	0.1	0.2
L-1200-1	D10	SNS-032 (BMS-387032)	0.1	-0.3
L-1200-1	D2	PD184352 (CI-1040)	0.1	0.9
L-1200-1	C9	Triciribine	0.1	0.1
		Seliciclib	0.1	0.0
L-1200-1	H1	Bosutinib (SKI-606)	0.1	1.3
L1200-3	F05	R547	0.1	-0.2
L1200-4	G10	AG-18	0.1	0.6
L-1200-1	D1	Selumetinib (AZD6244)	0.1	0.6
L-1200-1	C11	TGX-221	0.1	0.0
L-1200-1	F4	Vandetanib (ZD6474)	0.1	0.1
L1200-2	G11	SB590885	0.1	1.0
		Midostaurin	0.1	0.0
L1200-2	G06	BIX 02189	0.1	4.5
L-1200-1	G2	Erlotinib HCl (OSI-744)	0.1	0.5
L-1200-1	F9	Everolimus (RAD001)	0.1	-0.9
L-1200-1	G7	ZM 447439	0.1	0.8
L1200-4	B10	GSK2636771	0.1	2.0
L1200-2	A11	OSI-420	0.1	-0.1
L1200-4	F01	SAR131675	0.1	2.9
		Tivantinib	0.0	0.6
L1200-3	E03	PF-05212384 (PKI-587)	0.0	1.0
L-1200-1	A3	Imatinib Mesylate (STI571)	0.0	0.8
L-1200-1	A8	LY294002	0.0	0.4
L1200-4	E02	GW5074	0.0	5.6
L1200-3	E01	Palomid 529 (P529)	0.0	10.6
L1200-4	B03	ZM 323881 HCl	0.0	2.6
		NVP-BGJ398	0.0	-0.5
L1200-3	G10	Tofacitinib (CP-690550,Tasocitinib)	0.0	-0.2
L1200-4	G06	IPA-3	0.0	1.2
L-1200-1	H11	Regorafenib (BAY 73-4506)	0.0	0.7
L1200-3	H03	A66	0.0	1.0

L1200-4	D11	NSC 23766	0.0	-2.2
		VX-11E	0.0	-1.6
L1200-4	C11	Tyrphostin AG 1296	0.0	-1.3
L1200-3	G08	BGT226 (NVP-BGT226)	-0.1	-0.7
L1200-4	F09	EHop-016	-0.1	-0.7
L1200-3	E04	AS-252424	-0.1	3.0
L-1200-1	E1	BEZ235 (NVP-BEZ235, Dactolisib)	-0.1	0.1
L-1200-1	A9	JNJ-38877605	-0.1	-0.2
L-1200-1	D9	XL147	-0.1	0.6
L1200-2	G03	Ki8751	-0.1	1.5
L1200-3	G05	WAY-600	-0.1	-0.2
L-1200-1	H8	GSK690693	-0.1	0.0
		Doramapimod	-0.1	0.7
		Pilaralisib	-0.1	0.4
L1200-3	B11	AZD4547	-0.1	-0.1
L-1200-1	A4	Rapamycin (Sirolimus)	-0.1	0.0
L1200-3	A01	CAL-101 (Idelalisib, GS-1101)	-0.1	1.8
L-1200-1	A2	Cediranib (AZD2171)	-0.2	0.5
L-1200-1	E3	NVP-AEW541	-0.2	0.4
L1200-4	E10	SMI-4a	-0.2	-0.4
L1200-4	H10	CEP-32496	-0.2	-0.1
L1200-4	H01	Semaxanib (SU5416)	-0.2	2.7
L1200-4	E01	Torin 1	-0.2	3.1
L1200-2	H10	NVP-BHG712	-0.2	1.6
L1200-4	A04	Icotinib	-0.2	-0.6
L-1200-1	A7	KU-55933 (ATM Kinase Inhibitor)	-0.2	-0.1
L1200-3	F10	INCB28060	-0.2	0.0
L1200-3	G04	Trametinib (GSK1120212)	-0.2	0.0
L1200-2	B02	Amuvatinib (MP-470)	-0.3	2.3
L-1200-1	D5	GDC-0941	-0.3	-0.4
L1200-2	B03	Genistein	-0.3	4.0
L1200-3	A11	WP1066	-0.3	0.4
L-1200-1	H9	Alisertib (MLN8237)	-0.3	1.8
L1200-3	H09	Dinaciclib (SCH727965)	-0.3	0.6
L1200-3	F02	Phenformin HCl	-0.3	0.6
L-1200-1	G8	SGX-523	-0.4	0.2
L1200-4	B08	SKI II	-0.4	1.4
L1200-4	F05	CZC24832	-0.4	-0.1
L-1200-1	D7	MLN8054	-0.4	1.0
L-1200-1	G10	Roscovitine (Seliciclib,CYC202)	-0.4	-1.3
L1200-3	E11	GDC-0068	-0.4	-0.4
L1200-4	G04	Tofacitinib (CP-690550) Citrate	-0.4	3.1
L1200-3	E09	NVP-BVU972	-0.5	2.9
L-1200-1	A1	ETP-46464	-0.5	-0.5
L-1200-1	H10	SNS-314 Mesylate	-0.5	0.8
L-1200-1	E8	BI 2536	-0.5	-0.3
L1200-4	G11	GDC-0349	-0.5	-1.3
L1200-4	G02	PF-562271	-0.5	-0.7
L-1200-1	E7	Vatalanib (PTK787) 2HCl	-0.5	-0.3
L1200-4	H06	VE-822	-0.5	0.5
L1200-4	G08	Skepinone-L	-0.5	-0.6
L1200-4	C01	Tideglusib	-0.5	3.9
L1200-4	H08	AZD2858	-0.5	2.6
L1200-3	G02	TAK-733	-0.5	0.3
L1200-3	G11	BYL719	-0.5	-0.2
L1200-4	B02	IMD 0354	-0.6	1.4
L1200-4	E11	PRT062607 (P505-15, BIIB057) HCl	-0.6	-1.0
L-1200-1	H2	Gefitinib (ZD1839)	-0.6	0.5
L1200-4	H07	SSR128129E	-0.6	2.4
L1200-4	F11	Butein	-0.6	1.9
L1200-4	E04	Piceatannol	-0.6	0.8
L1200-2	A03	Zoledronic Acid	-0.7	1.3
L-1200-1	H7	GDC-0879	-0.7	0.0

L1200-4	H04	Fingolimod (FTY720) HCl	-0.7	1.1
L-1200-1	E4	Temsirolimus (CCI-779, NSC 683864)	-0.7	0.0
L1200-4	H02	NU7026	-0.7	3.0
L1200-2	A04	Pelitinib (EKB-569)	-0.7	1.5
L1200-4	H09	Sorafenib	-0.7	2.7
L-1200-1	H4	Y-27632 2HCl	-0.7	0.9
L-1200-1	G11	PD98059	-0.8	-0.3
L1200-4	H03	BAY 11-7082	-0.8	0.2
L1200-4	F04	SC-514	-0.8	-0.3
L1200-3	H01	Asiatic Acid	-0.9	1.7
		Refametinib	-1.0	1.1
L1200-3	F01	Degrasyn (WP1130)	-1.0	0.7
L-1200-1	H3	PI-103	-1.0	0.3
L1200-4	C05	Bardoxolone Methyl	-1.0	0.8
L1200-4	D03	GNF-2	-1.0	0.9
L1200-4	D09	ZCL278	-1.0	1.2
L1200-4	G09	TG003	-1.0	-1.2
L-1200-1	H5	ZSTK474	-1.0	0.0
		Rabusertib	-1.1	0.2
L1200-3	E10	TAK-285	-1.1	0.0
L-1200-1	F7	U0126-EtOH	-1.1	2.4
L1200-4	A01	Torin 2	-1.1	-0.7
L-1200-1	B8	OSU-03012 (AR-12)	-1.2	0.8
L1200-3	C01	Telatinib	-1.4	3.3
L1200-4	H05	XL388	-1.4	2.7
L1200-2	D06	Quizartinib (AC220)	-1.4	1.3
L1200-4	G05	IPI-145 (INK1197)	-1.5	-0.6
L1200-3	F09	Alectinib (CH5424802)	-1.6	1.1
L1200-3	G01	BKM120 (NVP-BKM120, Buparlisib)	-1.9	0.7

Table S2. Peptides tested as LIMK1 substrates.

	Name	Sequence	MW (Da)
1	Lyntide	EFPIYDFLPAKKK	1595.96
2	NUMBtide	RVVDEKTKDLIVDQT	1759.07
3	CDC25tide	ELMEFSLKDQEAK	1567.84
4	CDK2-pep	HHASPRK	831.96
5	IGF1Rtide	KKKSPGEYVNIEFG	1595.89
6	AP2M1tide	SQITSQVTGQIGWRREG	1903.19
7	CDK7-pep	NRAYTHQVVTRWYR	1850.14
8	CHKtide	KKKVSRSGLYRSPSMPENLNRPR	2701.25
9	DYRKtide	RRRFRPASPLRGPPK	1790.15
10	SGKtide	CKKRNRRLSVA	1329.63
11	Moesin-pep	NARDESKKTAND	1347.4
13	Histone-H3-pep	ARTKQTARKSTGGKAPGGC	1874.16
14	MYPT1-pep	RLGLRKTGSYGAL	1390.62
15	RS-pep	GRSRSRSRSRSR	1446.59
16	DAPKtide	KKRPQRRYSNVF	1577.85
17	PKC-pep	RFARKGSLRQKNV	1558.85
18	DYRK1-pep1	RARPGTPALRE	1222.41
